# Transfusion-Transmissible Infections among Voluntary Blood Donors at Wolaita Sodo University Teaching Referral Hospital, South Ethiopia

**DOI:** 10.1155/2016/8254343

**Published:** 2016-08-15

**Authors:** Fithamlak Solomon Bisetegen, Fanuel Belayneh Bekele, Temesgen Anjulo Ageru, Fiseha Wadilo Wada

**Affiliations:** ^1^School of Medicine, College of Health Sciences, Wolaita Sodo University, P.O. Box 138, Wolaita Sodo, Ethiopia; ^2^School of Public Health and Environmental Science, College of Health Sciences and Medicine, Hawassa University, Hawassa, Ethiopia; ^3^Wolaita Sodo University Teaching Referral Hospital Laboratory, P.O. Box 378, Wolaita Sodo, Ethiopia; ^4^School of Medicine, College of Health Sciences and Medicine, Wolaita Sodo University, P.O. Box 138, Wolaita Sodo, Ethiopia

## Abstract

*Background*. Transfusion-transmissible infections, human immunodeficiency virus, hepatitis B virus, hepatitis C virus, and syphilis are among the greatest threats to blood safety and pose a serious public health problem.* Objective*. To determine the magnitude of blood borne infections among blood donors at Wolaita Sodo University Teaching Referral Hospital.* Methods and Materials*. A cross-sectional study was conducted from 10/11/2015 up to 10/12/2015. 390 donors were consecutively included and data on donor's age, sex, blood type, and serum screening results were obtained by structured questionnaire and laboratory investigation. The collected data were entered into Epi Data version 1.4 and then exported to SPSS version 20.0 for analysis.* Result*. The seroprevalence of blood borne pathogens is 29.5% of which HCV, HBV, HIV, and syphilis account for 8.5%, 9.5%, 6.4%, and 7.5%, respectively. Multiple infections were observed among 2.8% of the infected individuals. In addition, age ≥ 30 has a significant association with HCV.* Conclusion*. Significantly higher prevalence of transfusion-transmissible infections was identified from blood donors and they remain to be the greatest threat to blood safety, so comprehensive screening of donors' blood for HIV, HBV, HCV, and syphilis using standard methods is highly recommended to ensure the safety of blood recipient.

## 1. Introduction

Although blood transfusion is one of the known therapeutic interventions that cut across a number of clinical disciplines, the practice is not without risks [1]. The highest risk groups are children suffering from malaria and anemia; women with pregnancy related hemorrhage; and victims of major trauma [2].

Transfusion-transmissible infectious agents such as human immunodeficiency virus (HIV), hepatitis B virus (HBV), hepatitis C virus (HCV), and syphilis are among the greatest threats to blood safety for the recipient and pose a serious public health problem [3].

Among HIV transmission ways blood transfusion accounts for 5–10% in Sub-Saharan Africa [4]. Similarly, 12.5% of patients who received blood transfusion are at risk of posttransfusion hepatitis [5].

The high prevalence of HIV, HBV, HCV, and syphilis has heightened the problems of blood safety in Ethiopia. Thus, continuous monitoring of the magnitude of transfusion-transmissible infections in blood donors is important for estimating the risk of transfusion and optimizing donor recruitment strategies to minimize infectious diseases transmission [3].

However, there is scarce published information about the burden of major transfusion-transmissible infections in the study area.

Morbidity and mortality resulting from the transfusion of infected blood have far-reaching consequences, not only for the recipients themselves, but also for their families, their communities, and the wider society [6, 7].

Only continuous improvement and implementation of donor selection, sensitive screening tests, and effective inactivation procedures can ensure the elimination, or at least reduction, of the risk of acquiring TTIs [8].

Evaluation of data on the prevalence of transfusion-transmissible infections, namely, HIV, HBV, HCV, and syphilis, among blood and plasma donors permits an assessment of the occurrence of infections in the blood donor population and consequently the safety of the collected donations. It also gives an idea of the prevalence of the transfusion-transmissible infections (TTIs) among blood donors which allows for assessment of epidemiology of these infections in the community.

## 2. Methods and Materials 

### 2.1. Study Area and Setting

Institution based cross-sectional study was conducted at Wolaita Sodo University Teaching Referral Hospital from 10/11/2015 up to 10/12/2015. The university hospital provides clinical services for 85,700 populations. In 2015, approximately 1,500 units of blood were transfused in the hospital.

### 2.2. Study Population

Three hundred ninety blood donors were prospectively recruited in the study from October 10, 2015, up to November 10, 2015, and convenient sampling techniques were used to recruit blood donors who were eligible to donation, consented, interviewed, and gave blood for serum screening of transfusion-transmissible infections.

All blood donors who fulfilled the national and regional blood bank criteria were included. Blood donors who did not meet the inclusion criteria (<18 years, >65 years, history of long-term medication use, and unwillingness to give oral informed consent) were excluded from the study.

### 2.3. Data Collection

Data on blood donor's age, sex, blood group, serological results of HCV, HBs Ag, HIV, and syphilis were collected at the time of blood collection, by using a structured questionnaire. The laboratory uses immune chromatographic techniques to screen blood donors. In addition, the blood samples were retested with ELISA technique to confirm the results.

### 2.4. Laboratory Testing

Five milliliters of venous blood was collected using sterile test tube from each blood donor. Serum was separated by centrifugation at a speed of 3500 revolutions per minute (rpm) for 5 minutes and 2 mL of serum was collected from each sample using sterile plastic vials. Blood group for each blood donor was determined at each study area using blood group antisera: anti-A, anti-B, and anti-D for Rh factor. Each donor was tested for HBs Ag and anti-HCV by ACON one-step insert rapid test strips in the laboratory. To confirm the results, samples were retested by using 4th-generation ELISA technique. Blood samples tested by using serological assays for HIV infection were screened by 4th-generation ELISA, Vironostika HIV Uni-Form II AG/Ab. Hepatitis B virus was screened by using an immunoassay ELISA Hepanostika HBs Ag Uni-Form II, hepatitis C virus by using the human anti-HCV 3rd-generation ELISA, and syphilis by using syphilis antibody ELISA (FTA).

### 2.5. Data Management

The collected data were entered into Epi Data version 1.4 and then exported to SPSS version 20 for analysis. Summary statistics such as frequencies and percentages were computed. The results were presented using tables, charts, and graphs. Chi-square was used to see a difference of blood borne pathogens between age, sex, and blood groups. A *p* value of <0.05 was considered as a significant difference.

### 2.6. Quality Assurance

All positive samples and 10% negative samples were retested in South Nations and Nationalities People Region/SNNPR/regional laboratory to ensure the quality. Standard operational procedures were strictly followed and QC materials were used for all serological tests. In addition, laboratory quality was assured by well-trained professionals, training, and supervision during sample collection.

### 2.7. Ethical Consideration

The study was carried out after getting approval from the ethical clearance committee of Wolaita Sodo University. Consent was signed before sample collection and the participants' information is used only for study purpose. Respondents were not identified by name and the participant had the right to discontinue the participation any time.

## 3. Result

### 3.1. Donor's Characteristics

A total of 390 blood donors were included during the study period; among these, 291 (74.6%) were male and 99 (25.4%) were female with female to male ratio of 1 : 3. The median age of the donors was 28 years with the range 18 and 60 years.

Regarding donors ABO blood group distribution, 200 (51.3%) of the donors were “O” blood type. The remaining 104 (26.7%), 76 (19.7%), and 9 (2.4%) of the participants were “A,” “B,” and “AB” blood types, respectively.

### 3.2. Prevalence of Blood Borne Pathogens

**Figure 1:**
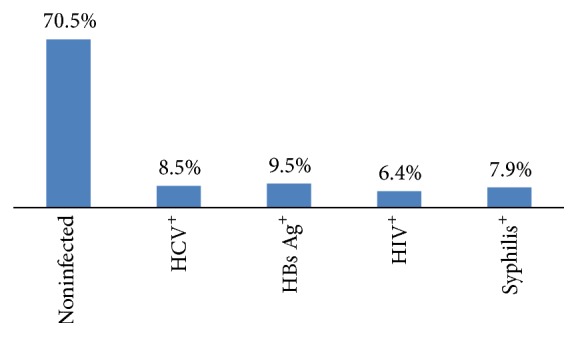
Distribution of blood borne pathogens infection among blood donors at Wolaita Sodo Teaching Referral Hospital 2015.

Blood borne pathogens were detected among 115 (29.5%) of the donors and 275 (70.5%) were free from the four infections. Donors with positive results for HCV and HBs Ag were 33 (8.5%) and 37 (9.5%), respectively. Twenty-five of them were positive for HIV whereas 31 (7.9%) were positive for syphilis test (Figure 1).

**Figure 2:**
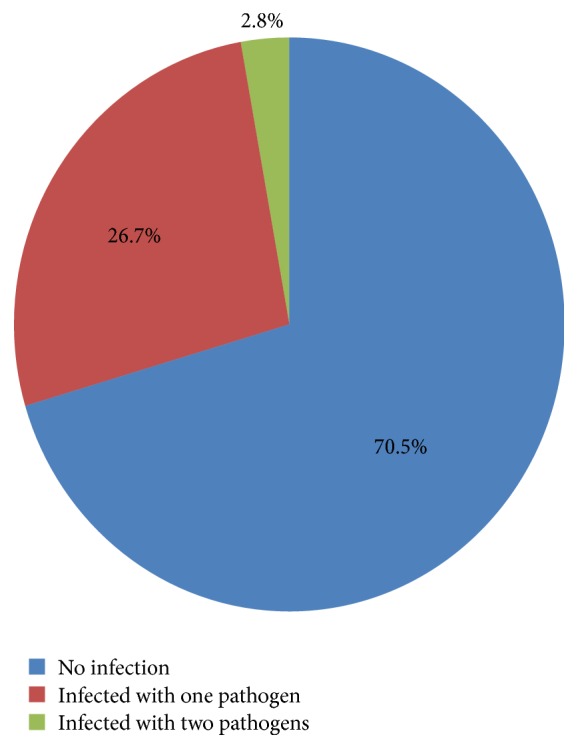
Infection status of blood donors by blood borne pathogens at Wolaita Sodo Teaching Referral Hospital 2015.

Among those who have the infection, 104 (26.7%) were positive for only one of the pathogens and the other 11 (2.8%) were coinfected with two of the four blood borne infections. Coinfection for more than two pathogens was not detected. Five of the coinfected donors were positive for HBs Ag and syphilis antibody tests. In addition, two donors with HIV positive screening result were also positive for HCV antibody test and other two were positive for syphilis antibody test (Figure 2).

Almost one-third of all male and 29 (29.3%) of all female donors in this study were positive at least for one of the four blood borne pathogens. Prevalence of HCV infection was 8.9% among males and 7.1% among female donors. Donors who are positive for HBs Ag test were 11.0% in males and 5.1% in females. About seven percent of the male donors and five percent of the female donors have been positive for HIV. Antibody evidence for* Treponema pallidum* was detected among 6.2% of male and 13.1% of female donors.

Regarding infection distribution with age categories, 65 (23.6%) of donors less than or equal to 30 years and 50 (43.9%) of donors greater than 30 years have evidence of at least one blood borne pathogen. The predominant positive result was for HBs Ag, which accounts for 24 (8.7%) and 13 (11.4%) among donors less than or equal to 30 years of age and greater than 30 years, respectively. Sex has no significant association with any of transfusion-transmissible infections but age ≥ 30 years has a significant association with HCV prevalence (*p* = 0.04) (Table 1).

Regarding the distribution of the infections with blood group, 8 (10.4%) of donors with A blood group are positive for HCV, whereas 10 (13%) of HBs Ag reactive donors were B blood group. Among A blood group donors, 17 (8.5%) were positive for HIV, and 10 (9.6%) were reactive for syphilis. RH^+^ donors showed that positive reaction for HCV accounts for 8.2% and 9.6% positive for HBs Ag. RH^−^ donors showed relatively high prevalence of HCV, 4 (11.8%), and syphilis, 5 (14.7%) (Table 2).

## 4. Discussion

In the current study, the seroprevalence of transfusion-transmissible infections was 115 (29.5%) which is similar to 29.85% reported in Burkina Faso [9], 21.6% in Cameroon [10], and 19.3% in Nigeria [11]. But the result is lower than 43.2% reported in Bahir Dar, Ethiopia [2], which could be due to the fact that most of the study participants in the previous study were commercial blood donors whereas only voluntary donors were included in this study. The current finding is much higher than previous studies conducted elsewhere, 14% in Islamabad [12], 9.6% in Sudan [13], 2.6% in Northeast Ethiopia, Gondar [3], 2.22% in Karnataka, India [14], 1.7% in Nepal [15], and 1.1%, 1%, and 0.93% in India [16–18]. Hence, even when compared with other resource constrained settings, the burden of blood borne pathogens is quite high in this study. Widest difference in blood borne pathogen in different studies in our country and others could be due to the use of different generation of ELISA test kits having different sensitivities and specificities and geographical factors.

Donors with positive results for HCV, 33 (8.5%) in this study, are comparable with 13.3% reported in Bahir Dar, North Ethiopia [2], 8.7% in Burkina Faso [9], 8.34% in Islamabad [12], and 8% in Ghana [19]. But the result is considerably higher than studies conducted elsewhere, 4.8% [10], 3.4% [13], 1.7% [20], 1.1% [21], 0.51% [22], 0.48% [23], 0.21% [24], 0.2% [17], 0.16% [25], and 0.03% [14]. The higher prevalence of this study as compared with the above findings could be supported by the reason that most of the donations before six years were commercial donors and they were donating blood without being screened for HCV and these strains may be circulating in the community without noticing.

Prevalence of HBs Ag among blood donors in this study, 9.5%, is lower than a prevalence of 25% in North Ethiopia [2], 14.9% in Burkina Faso [9], and 14.3% in Jos, Nigeria [26]. But it is in harmony with a prevalence of 10.1%, reported the Cameroon study [10]. On the contrary, the current finding is in discordance with studies conducted in Ethiopia and elsewhere, 6.2% in Northwest Ethiopia [20], 5.3% in Nigeria [21], 5% in Sudan [13], 4.7% in Gondar [20], 3.91% in Islamabad [12], 1.16% in China [22], 0.9% [18], 0.63% [17], 0.49% [24], and 0.3% [25] in India, and 0.09% [23] in Canada. These variations could also be due to actual changes in population risks or effectiveness of donor screening measures.

HIV prevalence of 6.4% observed among blood donors at Wolaita Sodo University Teaching Referral Hospital is lower than 11.7% for Bahir Dar, Ethiopia [2], but considerably comparable with 4.1% reported in Cameroon [10] and 3.8% reported in Ghana [19] but higher than previous studies conducted in the country and elsewhere [12, 13, 17, 18, 21–23, 25, 27, 28] in different countries. HIV prevalence in the current study shows high discrepancy with the nationwide adult HIV prevalence estimate of 1.1% [29], but lower than 10.2% reported in SNNPR [30]. The high prevalence of HIV seropositivity in comparison with the national figure and other local regions could be due to lower voluntary counseling test coverage in this region than the other [30].

Treponemal positivity prevalence of 7.9% in our study is lower than the prevalence of 13.5% reported in Ghana [19], but in harmony with 7.5% prevalence reported in similar study conducted in the same country, Ghana [31], and 5.7% in Cameroon [10]. Our finding is higher than 3.96% prevalence in Burkina Faso [9], 1.2% in Bahir Dar [2], 0.89% in Islamabad [12], 0.3% in China [22], 0.23% in India [28], 0.22% in Gujrat, India, and Uttarakhand, India [17, 18]. This could possibly be explained in association with the higher prevalence of HIV in this study where one could facilitate the transmission of the others.

In the current study, there was no association between sex, ABO blood group, RH factor, and any of blood borne pathogens. Age group ≥ 30 have a significant association with HCV which is unclear but could be explained by the fact that liver fibrosis increases with old age and in age groups 40–64 and could also be due to the highest seropositivity of HCV for individuals aged >30 [32, 33].

## 5. Conclusion and Recommendation 

In general, the prevalence of transfusion-transmissible infection is high in the study area. The blood donors in this study were voluntary subjects, who are apparently healthy, but this study found that these diseases are prevalent among volunteer donors. Thus, strict selections of blood donors with standard methods are highly recommended to ensure the safety of blood for the recipient.

## Figures and Tables

**Table 1:** Distribution of blood borne pathogens by sex and age among blood donors at Wolaita Sodo Teaching Referral Hospital, 2015.

Variable	HCV		HBs Ag		HIV		Syphilis	
	R	NR	R	NR	R	NR	R	NR
	No (%)	No (%)	No (%)	No (%)	No (%)	No (%)	No (%)	No (%)
*Sex*								
Male	26 (8.9)	265 (91.1)	32 (11.0)	259 (89.0)	20 (6.9)	271 (93.1)	18 (6.2)	273 (93.8)
Female	7 (7.1)	92 (92.9)	5 (5.1)	94 (94.9)	5 (5.1)	94 (94.9)	13 (13.1)	86 (86.9)

*Age category*								
≤30	22 (8.0)	254 (92.0)	24 (8.7)	252 (91.3)	11 (4.0)	265 (96.0)	13 (4.7)	263 (95.3)
>30	11 (9.6)	103 (90.4)	13 (11.4)	101 (88.6)	14 (12.3)	100 (87.7)	18 (15.8)	96 (84.2)

**Table 2:** Distribution of blood borne pathogens by donor's blood type among blood donors at Wolaita Sodo Teaching Referral Hospital, 2015.

Blood type	HCV		HBs Ag		HIV		Syphilis	
	R	NR	R	NR	R	NR	R	NR
	No (%)	No (%)	No (%)	No (%)	No (%)	No (%)	No (%)	No (%)
*ABO blood group*								
A^+/−^	5 (4.8)	99 (95.2)	7 (6.7)	97 (93.3)	5 (4.8)	99 (95.2)	10 (9.6)	94 (90.4)
B^+/−^	8 (10.4)	69 (89.6)	10 (13.0)	67 (87.0)	2 (2.6)	75 (97.4)	6 (7.8)	71 (92.2)
AB^+/−^	1 (1.1)	8 (98.9)	2 (2.2)	7 (97.8)	1 (1.1)	8 (98.9)	1 (1.1)	8 (98.9)
O^+/−^	19 (9.5)	181 (90.5)	18 (9.0)	182 (91.0)	17 (8.5)	183 (91.5)	14 (7.0)	186 (93.0)

*RH blood group*								
Positive	29 (8.2)	326 (91.8)	34 (9.6)	321 (90.4)	24 (6.8)	332 (93.2)	26 (7.3)	329 (92.7)
Negative	4 (11.8)	30 (88.2)	3 (8.8)	31 (91.2)	1 (2.9)	33 (97.1)	5 (14.7)	29 (85.3)
